# On the Nature and Treatment of Tic Douloureux, Sciatica, and Other Neuralgic Disorders

**Published:** 1844-07

**Authors:** 


### ART. IV.

*On the Nature and Treatment of Tic Douloureux, Sciatica, and other Neuralgic Disorders.* By H. HUNT, M.D.—*London*, 1844. 8vo, pp. 192.

WE have been much gratified by the perusal of this volume. Frequently, and strenuously, have we, in this journal, urged those who really have had experience to communicate its results;—to tell what they really do know; to enter minutely into points of treatment, and into the details of their own management of disease; to communicate freely for the common benefit what they have found most useful themselves. How excellent a book has Dr. Graves produced by doing this. He calls it a 'System of Clinical Medicine,' but happily it is no system at all. It is not a complete work on a science which is as yet only fragmentary and incomplete. It does not treat every disease as if it were thoroughly known, ab ovo usque ad mala. He does not seem to fear, lest he should by any possibility leave anything unsaid. But he gives the result of his own experience; the formulæ he employs, and the reasoning which he pursues whilst prescribing. Hence the practical usefulness of his book; the frequency it is recurred to in doubt and difficulties; the advantage so often found from his hints and excellent formulæ. A similar spirit pervades the writings of a physician, once so distinguished in the same city, which is especially remarkable for the abilities of its medical practitioners, the late Dr. Cheyne of Dublin. The few papers which he has left in the 'Cyclopædia of Practical Medicine' are full of the best practical matter. We especially remember those on gastric fever, wakefulness, and croup. We have read them often, referred to them still oftener, and always with benefit. No directions for the sick man's comfort ever seemed trivial to a man who was worked to death in consultation practice. The arrangement of the beds, pillows, coverlid, and the minutiæ of diet are not disregarded by a mind capable of the largest views of disease. The only drawback is that he wrote so little. To be sure, Dr. Cheyne and Dr. Graves are not ordinary men. But there are many excellent practitioners scattered over Great Britain who have made good use of ample opportunities of studying disease under the best circumstances for that study, those of undivided responsibility. And such might be of much use in their generation beyond their own sphere.

Dr. Hunt has done this with tic douloureux and neuralgic diseases. He informs us that he practised for some years in the south of Devon, where a warm and humid climate favorable to the development of the neuralgic habit gave him an opportunity of investigating this class of maladies. Finding that tic douloureux existed in very opposite states of the system; that it was cured by very opposite plans of treatment; that remedies at one time much vaunted, have fallen into disuse, and that those which cured one case were positively injurious in others,—he endeavoured to discover the causes of this difference. He examined every case on its own merits, scrutinising closely the various organs of the body as well as its general condition, and if he found any well-marked symptoms of disorder or disease of any important organ, regarded that as the exciting cause, and directed his remedies to its relief. This view of neuralgia is not a new one. Whytt viewed nervous diseases in the same way; but his list of causes is much too narrow, and his treatment, in consequence, is limited and defective. Dr. Hunt's view of tic douloureux is consequently comprehensive and thoroughly practical; and although he brings forward no specific, no new drug, he endeavours to lay down clearer indications for the uses of the old ones than has been yet attempted.

It may be said that there is some want of method and arrangement in the book, but the compensating advantage is that the author has not tried to systematise; that he has neither docked nor elongated his patient according to the length of his bed. But we proceed to our analysis.

*Description.* Dr. Hunt describes the character and peculiarities of tic douloureux clearly and forcibly. The pain, at first dull, then plunging, like electric shocks, with inconceivable rapidity through the different branches of the nerve, (usually the 5th and 7th, but occasionally others,) ceasing for a few minutes, to be renewed, until the patient is driven into a kind of frenzy, and gradually or abruptly subsiding. These paroxysms may be irregular or regular. "Those that come on suddenly and violently from the first are frequently the most severe, irregular, and obstinate." In proportion as the irregular form is prolonged, the attacks become more acute and frequent, and are brought on by trifling causes of irritation, as a touch, a movement, or sudden exposure to heat and cold. If depending on visceral disorder, the attacks vary with the primary disorder. The regular form recurs, with the regularity of an ague, at the same hour; at first trifling, increasing in severity to its acme, and gradually subsiding, several times a day, or daily, or with days' or weeks' intervals, but still so regularly, that the day or hour of its return can be foretold. There is a marked tendency in the disease to return annually at the same period in both forms. "Some are quite free from pain except during one, two, or three months in the year; others have their sufferings *much increased* in particular months, some asserting that they suffer most in October and November, and others in April and May."

It is instructive to trace the analogies in the different members of the same family of disease. As in epilepsy, an attack of tic is sometimes preceded by a feeling somewhat like the epileptic aura. In one case depending on disordered stomach and liver, the attack was invariably preceded by a sensation as if a fine thread passed from about the region of the pylorus through the chest and neck to the cheek when the pain began. In others, a sensation, as if a stream of cool air was gently passing over the part, preceded the pain: one gentleman compared it to the electric aura; others have remarked that for a few hours before the attack they have had that restlessness and irritability and incapacity to settle themselves to any occupation which is so well expressed by "fidgets." Dr. Hunt has found this state to prevail in cases depending on a disordered stomach. In one instance, the painful side of the face wasted, but in twelve months after the pain had been relieved, the wasted cheek had recovered its natural size. In Dr. Hunt's experience, the intensity of the pain has not depended, in the least degree, on the particular cause; there has been as much suffering when the cause was in disordered functions as from local organic irritation.

Dr. Hunt's division of this disease is entirely a practical one, according to the causes from which it originates, and these he arranges under nine heads :

| | | | |
|---|---|---|---|
| 1. | Tic Douloureux arising from the neuralgic habit. | | |
| 2. | „ | „ | dyspepsia. |
| 3. | „ | „ | dyspepsia, complicated with congestion of the liver and other viscera. |
| 4. | „ | „ | anemia. |
| 5. | „ | „ | morbid action in the spine. |
| 6. | „ | „ | disorder of the uterus. |
| 7. | „ | „ | disease of the brain. |
| 8. | „ | „ | local mechanical causes. |
| 9. | „ | „ | malaria, recession of eruptions, and other causes. |

1. *Tic douloureux arising from some peculiarity of constitution, or neuralgic habit.* Some individuals, especially women, are, it is well known, morbidly sensible to every impression, moral and physical; pleasure and grief, joy and anxiety, as well as more material causes which directly act upon the nervous system, as mechanical injuries, change of weather, unwholesome or improper food, stimulants and medicines, produce upon their delicate organization much stronger impressions than on ordinary persons. Dr. Hunt seems to assume that this delicate state of the nervous system is the cause of tic, but, as this peculiar constitution is very common and tic douloureux rare, it need not be said that this is but a predisposing cause. Practically, however, and Dr. Hunt's is eminently a practical book, this condition may be assumed as the cause; for the treatment very much consists in improving this state, and giving strength to the nervous system.

Such individuals are, says Dr. Hunt, greatly benefited immediately by a removal to a high, dry, and open situation, care being taken that it is not too bleak. A few months' residence in a mountainous region, such as North Wales, gives tone and strength rapidly, but the caution is requisite, lest, under the immediate feeling of improvement, the invalid is tempted to take an imprudent amount of exercise, which is often followed by exhaustion. From Dr. Hunt's experience, a humid, relaxing climate is very injurious to all this class of delicate females, "few things operating more detrimentally on their general health, or having greater power of producing that peculiar kind of constitutional disorder of which neuralgia is the frequent consequence."

As a general proposition, we are inclined to agree with Dr. Hunt, but here a rule which he himself so wisely lays down, must be attended to, that each case must be studied and treated according to its own nature and peculiarity. We have several patients now, in our eye, with delicate nervous organizations, who are more comfortable and in better health, in a warm and somewhat humid climate, than in a cold and dryer one. They are better also when the weather is damper and warmer, that is, when the peculiarities of the climate are more marked. These are exceptions, but not very uncommon ones. Celsus makes a good practical observation, that change to a worse air is often useful. Where neuralgia has been brought on in a warm and relaxing climate, the change to a dry and colder one should be tried. But, on the other hand, those with a neuralgic habit of body, who are living in a dry, cold, and inland situation, may find benefit by a change to a warmer and more humid residence near the coast.

The injurious effects of anxiety, worry, and other moral causes, need hardly be pointed out. We recollect hearing of a physician of celebrity, who would not continue to attend any patient who had any weight upon his mind: probably from feeling that his visits were useless. In this state of the nervous system, trivial causes, as Dr. Hunt says, are " the greatest obstacles which we have to encounter in the treatment." In the treatment of these patients it should be remembered, says Dr. Hunt, that they " cannot bear harsh or violent remedies with impunity, but that they require a steady and patient perseverance with mild ones, until a more healthy action is induced ; afterwards, by a cautious use of tonics, combined with sedatives, and attention to a proper plan of living, the general strength may be so increased, that the neuralgic habit will at length, to a certain extent, be overcome." Caution is necessary, adds Dr. Hunt, lest the violence of the pain may lead to the exhibition of powerful tonics and narcotics by which the neuralgic habit may be confirmed. In addition to the common rules, to use exercise to produce such an amount of fatigue as moderate rest will recover, and to rest during digestion, Dr. Hunt insists on the necessity of feeble persons avoiding exercise for some time previous to their meals, for if they expend their nervous energy just before eating, digestion will be impeded. He gives a case of a gentleman subject to tic, who was in the habit of taking violent exercise in shooting, hunting, &c., who could never dine before he had rested quietly nearly two hours, for if he did, a violent paroxysm of tic was the certain consequence. And what is curious was, that the tic depended on an organic cause. These rules are particularly to be observed by those in whom fatigue after exertion is increased by some or other stimulants. It is " a feverish fatigue," and it is removed by a saline draught with ammonia, or a few grains of nitrate of potash and sal volatile.

These feeble persons are often much fatigued by dressing, lose their appetite for breakfast, and beginning the day badly, go through it tired and weary. A little nourishment, such as a cup of coffee, with a little bread, before leaving bed, enables them to dress with comfort, to sponge with cold water, or, what is better, to use the shower-bath and friction. Most fully do we agree with Dr. Hunt as to the importance of these seemingly trivial hints.

2. *Tic douloureux from dyspepsia.* The neuralgic habit of body may be the effect of previous disease. In investigating the history of cases of tic, Dr. Hunt has found that the disorders which have preceded the pain, and which may be regarded as its cause, are such as debilitate the body, either suddenly or by slowly undermining its powers. Of the former, influenza, as Dr. Holland pointed out, was a frequent cause. Of the latter, disorders of the digestive organs, of an atonic or nervous character, rather than of an inflammatory, are the most common. Indeed, however various the causes, the stomach is usually the first organ that suffers, although the patient may be unaware of it, and expect that he is quite well, excepting the pain in his face : a remark of Dr. Hunt's which is applicable to many diseases.

Two cases are detailed of tic arising from the simplest form of stomach disorder.

A middle-aged dean, a generous liver, finding himself growing stout, adopted a spare diet, with excessive walking exercise, which brought on general debility, indigestion with acidity, and, in a few weeks, constant pain in the cheek, aggravated in paroxysms. An emetic, followed by a warm purgative, and a course of liq. arsenicalis, combined with a sedative, relieved him.

The second case was that of a strong, athletic man, of forty, whose diet for many years had been a beefsteak for breakfast, with coffee, a full allowance of meat with two thirds of a bottle of port for dinner, and the remaining third, with meat, for supper. He had intense neuralgia of the left cheek, the paroxysms returning at all hours both of the day and night. Tongue foul, appetite good, but uneasy feelings owing to digestion. An emetic relieved the pain at once. It was followed by warm aperients, and a more rational diet, and he was soon cured.—The rapid amendment in both these cases Dr. Hunt judiciously attributes to the simplicity of the cause. But such simple cases are rare. For many years he has made it a regular practice in these cases to begin with an emetic: much glairy and tenacious mucus is evacuated, which, he has not the slightest doubt, facilitates the action of the medicines, especially of arsenic. If the paroxysm is regularly intermittent, the emetic should be given an hour before the fit; and after the emetic, a warm aperient draught, of twenty grains of rhubarb and sulphate of potash, with thirty drops of sal volatile, in some aromatic water. After this, a course of arsenic, combining it with a sedative, or with a few grains of the bicarbonate of potash if there is an acid stomach, he finds the best remedy: beginning with four minims three times every day, with double the quantity of compound tinct. of camphor, and gradually increasing the dose of liq. arsenicalis, until there is some decided symptom of its action, which is commonly evinced when the dose has amounted to ten. When the pain has considerably decreased, he discontinues the medicine for a few days, and recommences with the original small doses, and finds it rarely necessary to increase them, but continues them for several weeks, if not months, after the pain is removed; for patients should be strictly cautioned against the error of thinking themselves cured as soon as the pain is relieved. They must persist in the medicine and diet until the tone of their stomach is quite restored, or the pain will return. The susceptibility of the stomach towards this remedy is various. In some cases it must be given on a full stomach. If the pain at any time increases, the aperient should be repeated, and the quantity of arsenic increased. During the whole treatment an occasional aperient is useful. When the pain is relieved and the stomach improved, the substitution of a grain of quinine, three times a day, is useful, although it would have disagreed at first. Large doses of iron, quinine, bark, &c. are injurious in these cases. The arsenic may disagree *immediately*, producing an indescribable sensation of distress in the stomach, dryness of the fauces, white tongue, and other symptoms of gastritis. It should be at once discontinued; and as this morbid sensibility is probably accompanied with slight inflammation of the mucous membrane, a rigidly farinaceous diet, in small quantities and lukewarm, should be ordered; with small doses of nitrate of potash, combined with two or three minims of Scheele's prussic acid, three or four times a day, and three or four grains of James's powder, at bedtime. If aperients are needed, a common lavement, a little castor-oil, or two or three ounces of Pulna water; a sinapism to the stomach, or rubefacient liniment, and an occasional blister. After a few weeks of this treatment, arsenic may be borne, and at first should be given with or just after meals; and if it again disagrees it should be abandoned altogether. It is more beneficial to do little than much in such cases; by being content with a proper diet, and by avoiding anything that can possibly irritate the over-sensitive nerves. The attention to diet in all cases of neuralgia from dyspepsia is most important; and Dr. Hunt gives the result of his experience.

In cases of extreme sensitiveness of the stomach, the mildest food is sometimes necessary. Such patients should live entirely on farinaceous food, until the nerves of the stomach become less sensitive; which is known by the tongue becoming cleaner, and the general feelings (better known to the patient than described,) returning to those of health. When the stomach has been thus improved, some animal food should be added, beef-tea, a chicken prepared thus: "a chicken is to be wrapt in muslin, and stewed for twelve or fourteen hours, with half an ounce of vermicelli and a few whole peppercorns, until the whole has become a jelly; some of this, diluted, if necessary, with a little toast, forms a very nutritious and easily-digested meal." As the appetite improves, a slice of chicken or game, with stale bread or toast, &c. In such weak stomachs every kind of food has a tendency to become acid, which is in some measure prevented by some slight stimulus with or after meals; a little weak brandy and water, or a teaspoonful of sal volatile in a glass of water, or a cup of coffee.

For those less sensitive, or whose morbid sensibility has been quieted, a plain nutritious diet, of animal food, (chicken, game, mutton, and venison are the best kinds,) with stale bread or toast, and with a few well-boiled vegetables, *if they can be taken with impunity;* and there is seldom any objection to plain puddings of rice, bread, and tapioca. All fish, pastry, rich puddings, fruit, new vegetables, pickles, cheese, and various sauces must be strictly forbidden.

Errors in quantity may be prevented by two rules: 1st, to live simply and to avoid a variety of dishes; and, 2d, to eat slowly, that the first indications that sufficient food has been taken may be felt and obeyed.

Water is the best drink. Some, especially those who have indulged in wine, require the stimulus of a moderate quantity in order to digest at all. But the smallest quantity for this purpose should be taken, and as strength is gained, this allowance should be gradually diminished and discontinued altogether. A little brandy and water or a glass or two of good sherry or sound port are the best. For breakfast, bread or toast, weak café au lait, or scalded milk, prepared by placing the can of milk on a stove moderately heated, until all the cream has risen to the surface, which is to be removed when cold. This is easily digested. Those who can digest bacon may eat it. It should be toasted after being boiled. The other hygienic rules laid down in the previous chapter should be followed.

As good rules for the treatment of atonic dyspepsia are useful in so many chronic diseases, and disorders of the general health with which it is associated, we need not apologize for quoting so largely; for on its right treatment much of the success of a physician depends. The chief point on which we are disposed to differ with Dr. Hunt is in the use of vegetables and of farinaceous puddings, in addition to meat. Except in those comparatively few cases where the mucous membrane is so sensitive that it will not bear meat at all, we find that on the whole it is better to limit our patients to a dinner of meat, stale bread or biscuit, and rice. Vegetables, especially potatoes, are often injurious; and a patient who likes them is not the best judge if he is the exception; and if farinaceous puddings are allowed in addition to meat there is danger of error in quantity. The difficulty, we well know, in limiting the diet is to get our directions attended to.

3. *Tic douloureux from dyspepsia, with congestion of the liver and other viscera.* The symptoms, in addition to the local pain, are "a sallow, muddy complexion, with eyes half jaundiced; foul tongue; hard and tense abdomen; slugglish, irregular bowels; and urine scanty, high coloured, or turbid."

Large doses of opium, taken to mitigate the pain, often act injuriously, by weakening the stomach and producing this congestion.

Long-continued anxiety Dr. Hunt regards as one of the most frequent causes of it, as well as sedentary habits, too great exertion of mind or body, a residence in malarious districts. But he has somewhat strangely omitted what must be as material as any of these causes—errors in the quality and quantity of the food, of long continuance. Even long-continued anxiety and sedentary habits would have much less effect in producing this condition if the indvidual accommodated his diet to his diminished powers. From our own experience, we think very few persons are fully aware, who are in these circumstances, how much their bodily comfort is dependent on the strictest attention to the quantity and simplicity of their food, and especially, perhaps, when the injurious effects are produced some time after a meal.

The treatment Dr. Hunt adopts in these cases is to unload the congested organs by an emetic, followed by brisk mercurial purgatives, until the "system is unloaded, and the viscera are stimulated to a more healthy action." In old cases this requires perseverance and time; and, when the congestion is obstinate, a visit to Carlsbad, to ensure a course of saline aperients, change of air, habits, &c. Kissingen, however, is more conveniently got at. The great disadvantage, however, of German spas is their unwholesome table d'hôtes, especially in such cases as these, where diet must be most important. Fortunately we have two excellent saline springs at home, Leamington and Cheltenham.

It is in these sort of cases, Dr. Hunt observes, that purgatives of croton oil are beneficial; and he thinks this is especially the case when it acts as an emetic as well as a purgative. Mercury, given so as to affect the system, should, as a rule, be avoided in disorders of the nerves; but in these cases, when obstinate and of long standing, it may be necessary.

When this congestive condition is removed, tonics or sedatives may be employed. When the pain has been excessive and exhausting, Dr. Hunt gives sedatives, in doses of sufficient strength, repeated at short intervals. He has found belladonna the most efficacious, particularly when the pain is irregular. When regularly intermitting, he prefers a grain or two of solid opium, with camphor, an hour or two before the expected attack. This plan must be modified in those of feeble powers, in whom, with the congestion, there is much debility and irritability: milder purgatives, with as much nourishment as can be borne, and the free exhibition of cordials and perhaps sedatives. As the secretions improve, tonics, combined with aperients, gradually increasing the former and decreasing the latter. When the tongue becomes clean and the complexion clearer, if the pain continues, arsenic is a useful tonic, with a sedative (opium, belladonna, or conium,) at night, to ensure rest, and smaller doses during the day, to allay irritation. And even when the pain is overcome, attention is required to strengthen the system, to prevent a relapse, by quinine, steel, &c., continuing the plan for many months—avoiding the common error of attempting to cure quickly by large doses of the tonic.

4. *Tic douloureux from anemia.* Where there is no local cause of irritation to account for the pain, nor the neuralgic habit, but where persons, naturally of a strong constitution, have a pallid skin, loss of strength, and often symptoms indicating a deficiency both in the quantity and quality of the blood, Dr. Hunt arranges the cases under this division. It is this class of cases which are so much benefited by iron, continued unremittingly for months, until there is evidence of pure red blood in the system. In one case, which is detailed, of this kind, where the paroxysms were very intense, a grain of belladonna was given during the attack, and ordered to be repeated, if necessary, for three successive hours. Soon after the third pill was taken, the pain began to subside. The pain did not return for a week, and then one grain checked it. In these cases, belladonna is often very serviceable in allaying the general irritation of the system, as well as in checking the pain.

5. *Tic douloureux from morbid action in the spine.* Three or four cases of very obstinate tic douloureux of the face in females fell under Dr. Hunt's care, in which he could not discover any local cause, but attributed the disease to a deranged state of the general health. All plans of treatment and every kind of medicine failed to afford relief. Some symptoms, such as loss of power in the legs, led to an examination of the spine; much tenderness was discovered on pressing some of the vertebræ. Caustic issues were used, and the recumbent posture enjoined, with complete relief to the tic, and restoration or improvement of the general health. The duration of this treatment varied. The connexion between the state of the spine and the pain in the face could not be doubted. "Whether it originated in that part," adds Dr. Hunt, "may be doubtful." Whilst admitting fully that much injury was done ten or twenty years back by treating the tender spine of hysterical females by cupping, issues, and confinement to the horizontal posture, instead of strengthening the system, Dr. Hunt thinks that the opposite error is fallen into now, "with far more serious results;"—examples having occurred in his own practice and in that of others, "of protracted misery or death" produced by treating organic disease of the spine, its cord, or envelopes as a functional hysterical affection, requiring pure air and tonic remedies; and a careful attention to such cases has convinced him that the pain in the spine, which at first was purely hysterical, depending on some deranged state of the general health, degenerates gradually into real disease of structure. The commonly-admitted explanation of this, that nervous excitement leads to increased vascular action and a low kind of inflammation, he illustrates by referring to those cases of tic where the violent paroxysms of pain are, after some time, followed by great and constant tenderness of the cheek, and constant swelling, as if from infiltration of serum. It is a point of great practical importance to discover when this transition takes place, and it is one of difficulty, as real disease may exist and be masked by hysterical symptoms. Dr. Hunt alludes to the difficulty, but attempts to lay down no rules for our guidance. There is a middle treatment, which we have not seen in books, but which we have employed, not indeed in tic, but in cases where there was long-continued tenderness of the spine, with disturbance of the general health, and various nervous symptoms, not yielding to the ordinary means. This is, a caustic issue or seton over the painful spot in the spine, combined with moderate exercise, and other general means calculated to improve the health. By this plan, the most injurious part of the old treatment—confinement to the recumbent posture—is avoided.

We rather demur to any direct comparison between the unfortunate results of treating hysteria as organic disease, and the opposite error to which the times are more prone. Death may not have been the consequence of the first mistake, but what a multitude of female constitutions has not this "nimia diligentia" seriously impaired, rendering them unfit for the proper discharge of the active duties of the remainder of their lives, and often entailing a similar condition of body upon their half-organized offspring! Still the error of treating organic mischief as mere hysteria is a very serious one. It brings us back to that truth, which we cannot too steadily remember, that "every case of disease is a separate study," and that no amount of experience will justify routine practice.

6. *Tic douloureux from disorder of the uterus.* Four cases are detailed. In all, the original cause of ill health appeared to be a bad labour attended with flooding, and followed by menorrhagia and leucorrhea. In two, the pain commenced soon after confinement, and always returned at the menstrual periods. In the other two the pain commenced several years after confinement, but this was evidently the cause of their broken health; in one of these as the disordered uterus was cured, so was the tic, in the other, the uterus was not relieved, neither was the neuralgia.

In all of these, carbonate of iron had been taken in considerable quantities, before Dr. Hunt saw them. The weakness and bloodless countenance seemed to indicate it, but it failed by increasing the menorrhagia, and, consequently, inducing greater debility. In these cases, Dr. Hunt says, arsenic is peculiarly adapted: he thinks it subdues morbid sensibility of the nerves, and restrains passive menorrhagia. He has also found belladonna and opium, previous to the menstrual periods, useful. Cold astringent injections to the uterus and cold hip-baths, sometimes saturated with bay-salt, are valuable adjuvants. The clothing over the loins should not be too warm, and lounging on warm relaxing couches should be avoided. The possibility of the menorrhagia depending on a loaded state of the bowels should not be overlooked. To show the connexion between the uterus and neuralgia of the face still further, one case is reported in which a lady was subject to neuralgia of her face in all her pregnancies, at about the same period. It lasted about ten days, and was cured by arsenic. In another case, a paroxysm like tic came on before delivery; it was very severe during delivery, and subsided as soon as the child was born. The placenta was retained, so that extraction was necessary; as soon as the fingers reached the uterus, the pain returned, but ceased as soon as the placenta was removed, and did not return. Allusion is made to the face-ache, which always precedes menstruation in some delicate young women; and a case is given, in which a lady had severe neuralgia of the ulnar nerve, coming on when she was about four months advanced in pregnancy, and continuing until her confinement, after which it immediately ceased.

When there is a periodical return of tic every month, Dr. Hunt has found that it was connected with some irregularity in menstruation.

7. *Tic douloureux from disease of the brain.* In these cases the pain may be at first intermittent, but by degrees the attacks become more constant and irregular, and confusion of thought, loss of memory, impairment of vision, difficulty of articulation, tottering gait, and palsy by slow degrees, indicate the cause. There is nothing new under this head.

8. *Tic douloureux from local mechanical causes.* The connexion between the pain and the teeth, to which the patient so often refers his sufferings, should be carefully examined. It is useless to make the examination during the paroxysm. But if, in its absence, any tooth on pressure, or on being slightly struck with a metallic instrument, is sensitive, or the paroxysm is brought on, the tooth should be removed. In all cases decayed teeth should be extracted or stopped, and stumps removed. Several cases are given of tic from diseased teeth, where either exostosis of the end of the fang, or a rough state, or the periosteal covering converted into a lamina of bone was discovered. Dr. Hunt has remarked that decay of the teeth was more frequently the consequence than the cause of tic; the teeth which were good and firm originally, becoming affected with caries at that point at the edge of the gums, where the enamel terminates. Under this head, unnatural growths of bone, local injuries of nerves, foreign substances pressing upon nerves, and the diseased state of nerves in the stump after amputation, are alluded to among the occasional causes of tic; and the propriety of keeping the general health as good as possible by giving tone and strength to the system, and by allaying irritation, insisted on even in those cases where the cause of irritation cannot be removed.

Dr. Hunt mentions malaria as a common cause of tic, but thinks that Dr. Macculloch has so ably treated that subject as to preclude the necessity of anything further than an allusion to it.

*Periodical headach.* Brow-ague, as it is commonly termed, only gradually assumes a periodic character, beginning as diffused headach, coming and going at uncertain times, at length returning with more severity at particular hours, and assuming the quotidian and more rarely the double quotidian or tertian type. The fully formed paroxysm commences with uneasiness in the temple, gradually increases for an hour or two, until it becomes excruciating pain which continues for an hour or more, and gradually subsides, leaving the individual perfectly easy in the interval. It often arises from malaria; sometimes from mere debility, or from anxiety, over-nursing, or any other cause of exhaustion. Two cases are given, in which it returned annually at the same time, one in August, the other in April. Dr. Hunt commences the treatment by an emetic, followed by a purgative, after which a course of quinine or arsenic, continuing the remedy for two or three days after the pain has ceased, and repeating it for a few days at the end of a week, otherwise, as he has found, the pain is very apt to return about the tenth day. In some cases, capsicum or black pepper, combined either with the quinine or arsenic, cures more quickly than either used separately. He prefers one or two grain doses of quinine three times a day, or every six hours, to larger doses.

*Sciatica.* Painful affections of this nerve, though classed under the same name are very various.

Acute inflammation of the nerve is most frequently the effect of exposure to wet and cold, standing or sitting in water, or wet ground, in boats or in coaches. The pain in the course of the nerve is very severe, aggravated by the slightest motion, and attended with high febrile symptoms. It requires strictly antiphlogistic treatment. Cupping along the course of the nerve, brisk purging with calomel, followed by salts and senna, each dose containing thirty drops of vin. colchici, or three to six grains of powdered colchicum, every five hours, until the febrile symptoms are diminished. Then ten to fifteen grains of Dover's powder will greatly relieve the pain; for the pain is seldom reduced in proportion to the reduction of the febrile symptoms, a point which should be remembered. At this stage, two grains of calomel, from four to six of powdered colchicum, and five of Dover's powder, should be given every six hours, with some diuretic, and an extra dose of opium at night if necessary; repeating the black draught every second morning. It is needless to add that the effect of the colchicum should be watched. Even after these means, the pain may be obstinate and severe, requiring blistering. It may be necessary to continue the calomel until the gums are sore, as it is important to prevent the disease from becoming chronic, which it is very prone to do.

This chronic inflammation of the nerve is obstinate, and sometimes followed by loss of power, wasting and shrinking of the limb. The pain does not wholly subside, but is aggravated in paroxysms, and particularly at night. In some cases there is nocturnal fever. To distinguish this state from one of pure neuralgia, is often difficult. Nocturnal fever, and scanty, high-coloured urine with sediment, sometimes assist the diagnosis. If the patient has this chronic form of the disease, when first seen, purgatives should be given, especially if his age is advanced. Calomel and colocynth, followed by castor oil, sometimes bring away hardened feces, and occasionally perseverance in the purgatives has relieved the disease, which seemed to depend on a loaded state of the bowels; but in general mercury will be necessary. The following formula Dr. Hunt employs—

℞ Hydrargyri phosphatis, gr. j.

Opii, gr, j.

Antim. potassæ tart. gr. $\frac{1}{6}$.

Fiat pilula omni nocte sumenda.

If there is much nocturnal fever, in addition to this, moderate doses of nitrate of potash and colchicum should be given three times a day, with occasional aperients. If much exhaustion of strength and emaciation, the compound decoction of sarsaparilla with the liquid extract during the day, and the mercurial pill at night. Counter-irritation is especially useful. Open blisters, the tartar-emetic plaster, or even a caustic issue behind the trochanter. Dr. Hunt alludes to the hydriodate of potash as producing a powerful effect in some cases, but says he has not himself used it.

Sciatica, of which pain is the only symptom, returning in paroxysms like electric shocks, is occasionally met with, and should be treated like pure neuralgia, first by purgatives, and then by steel, quinine or arsenic with sedatives.

A case is given in which sciatica existed with piles and prolapsus ani, and the removal of the piles relieved the pain.

Persons subject to sciatica should wear flannel drawers next to the skin, and if they are very susceptible to weather, washed-leather drawers over the flannel, during the winter months. We have seen benefit in some cases of obstinate pain in the sciatic nerve from washed-leather drawers worn next to the skin.

It is well known that intermittents may become remittents. Dr. Macculloch alludes to the explanation which has been given, that this is owing, in intermittent fevers, to some intercurrent visceral derangement, but thinks there is no decided proof. Dr. Hunt has found that cases of intermittent neuralgia which have become remittent, have again resumed the intermittent type, after an emetic and brisk purging or after the system has been brought under the action of mercury; and he is inclined to believe that when an intermittent neuralgia becomes remittent, it is to be attributed to some disorder of some of the viscera. Practically this is important,—the change indicating the necessity of evacuants and deobstruents, after which quinine may again be given.

Dr. Hunt gives another hint worth attending to; that when tic occurs annually, it depends on some deviation from health, to which many persons are annually subject, and that by attending to the first symptom of this general disorder, you anticipate the local disease.

The volume concludes by some observations on the use of sedatives and tonics, in neuralgia.

*Sedatives.* There are three modes of giving sedatives to allay violent pain.

1st. By a very large dose at once. This is hazardous.

2d. By a smaller one, gradually increased. This is uncertain.

3d. By giving the largest ordinary dose, and repeating it every hour, or every second hour. This is the plan Dr. Hunt adopts. He is most partial to belladonna, and the largest quantity he has given has not exceeded one grain for three successive hours. Before the third dose is given the patient should be visited. When a decided check has thus been given to the pain, smaller doses, Dr. Hunt finds, keep both the pain and morbid irritability under control,—a third, a half, a grain once, twice, or three times every day, gradually diminishing the dose as the case improves. The patient should be instructed to take a dose at any time when the pain threatens to return. As a general rule, sedatives should not be given on the first visit, as by obscuring the symptoms, they render the detection of the cause more difficult. When there is uterine irritation, they are beneficial during menstruation, and when menorrhagia also exists, if given a few days before the commencement of menstruation, they often both moderate the discharge and allay irritation and pain. In these cases a mild aperient, such as castor oil, compound decoction of aloes, with or without gray powder, or blue pill, should precede the sedative. In cases where there is pain in the spine arising simply from weakness and irritability, sedatives are useful with quinine and steel; but when complicated with visceral obstructions, or of long standing, and when it has become a fixed disease, little relief can be expected.

*Tonics.* Arsenic is clearly Dr. Hunt's favorite remedy, and he devotes a chapter to the rules which guide him in its administration. He finds it act most favorably on those of lax fibre, languid circulation, cold and moist skin, and whose urine is pale and plentiful. In such it not only relieves the pain, but gives general strength. Where the urine is high coloured and scanty, with lithate of ammonia sediment, the tongue loaded, and especially its tip and edges red, it disagrees and aggravates the pain; but it often is useful when the visceral disorders on which these symptoms depend are removed.

It is peculiarly appropriate when the disease arises from malaria and is strictly intermittent, when it depends on a neuralgic habit, disorder of the uterus; but when tic is associated with morbid action in the spine, or with anemia, or is complicated with visceral congestion, it is usually injurious.

Dr. Hunt does not doubt that many will think he has laid too much stress on the necessity of unloading the bowels and producing a more healthy secretion before tonics or sedatives are given. We are not of this number, as we are convinced that it is a matter of primary importance, and that it cannot be too strenuously insisted on. The combination of tonics with aperients, (as quinine with sulphate of magnesia and sulphuric acid,) which is often good practice, Dr. Hunt has not dwelt on as we think it deserves.

Arsenic should not be given for many months successively, but should be discontinued from time to time, as soon as any of its peculiar effects on the system are discovered, and not resumed until all symptoms of its action have subsided. These are, less plentiful secretion of urine, more acid and high coloured, with at last deposition of lithate of ammonia, disinclination to food, rather hot skin, sensation of general warmth, with a tingling in the fingers and toes; but all these symptoms seldom occur together. The change in the urine alone is a sufficient indication that the arsenic should be discontinued. Arsenic has been given in the solid form. But we refer our readers on this point to the book itself, for we should not feel justified in giving less than every word of caution with which the recommendation under any circumstances of such a dangerous remedy, was guarded. Dr. Hunt evidently puts but little faith in local remedies of a sedative kind. He has found aconitine ointment (one grain to a drachm) of temporary benefit, as well as belladonna, and he has often used with advantage a poultice made of equal parts of linseed meal and bruised stramonium seeds.

The length and completeness of this analysis afford sufficient proof of the estimation in which we hold the work itself. No man could have written it unless he had seen much disease and carefully studied it. The author's judgment and his principles of investigation are sound. He studies every case on its own merits, and makes his facts available as guides in future practice by examining, comparing, and arranging them. We can confidently recommend this volume to every student of disease ; a large class; for what practitioner does his duty who is not a student all his days?

